# Comparative evaluation of the Mayo Clinic Florida microdosimetric kinetic model and mMKM for carbon ion treatment planning: A matRad‐based analysis

**DOI:** 10.1002/acm2.70645

**Published:** 2026-05-31

**Authors:** Shannon Hartzell, Sridhar Yaddanapudi, Keith M. Furutani, Alessio Parisi, Xiaoying Liang, Chunjoo Park, Bo Lu, Jun Tan, Lisa Seckler, Remo Cristoforetti, Taku Inaniwa, Niklas Wahl, Chris J. Beltran

**Affiliations:** ^1^ Department of Radiation Oncology Mayo Clinic Jacksonville Florida USA; ^2^ Department of Medical Physics in Radiation Oncology German Cancer Research Center – DKFZ Heidelberg Germany; ^3^ Heidelberg Institute for Radiation Oncology (HIRO) Heidelberg Germany; ^4^ University of Applied Sciences (THM) Giessen Germany; ^5^ Faculty of Physics and Astronomy Heidelberg University Heidelberg Germany; ^6^ Department of Accelerator and Medical Physics Institute for Quantum Medical Science National Institutes for Quantum Science and Technology Chiba Japan; ^7^ Department of Medical Physics and Engineering Graduate School of Medicine Division of Health Sciences Osaka University Suita Osaka Japan

**Keywords:** carbon radiotherapy, mayo clinic florida microdosimetric kinetic model, modified microdosimetric kinetic model, RBE modelling, relative biological effectiveness

## Abstract

**Purpose:**

Modeling relative biological effectiveness (RBE) is central to carbon ion radiotherapy treatment planning. The modified Microdosimetric Kinetic Model (mMKM) is a clinically established RBE framework that has guided treatment protocols at many existing carbon centers, while the Mayo Clinic Florida Microdosimetric Kinetic Model (MCF MKM) is a recently developed alternative. This work aims to implement the MCF MKM in the open‐source treatment planning system *matRad* and to quantitatively compare its RBE‐weighted dose predictions with those of the clinically established mMKM using identical physical dose distributions across multiple disease sites. These findings will help assess their dosimetric equivalence and inform protocol development for carbon ion radiotherapy at MCF.

**Methods:**

Monte Carlo simulations of the MCF carbon beamline were performed to generate physical (IDD, LET, lateral spread) and biological base data for integration of each RBE model into *matRad*. Treatment plans were generated for six patients, each corresponding to a different disease site, using clinical beam configurations with carbon‐reference dose prescriptions, and plans were optimized using the MCF MKM. To isolate differences attributable solely to the RBE model, the resulting physical dose distributions were held fixed and RBE‐weighted doses were recalculated using the mMKM. Dose volume histogram (DVH) metrics and spatial dose‐difference maps were used to compare target coverage and organ‐at‐risk doses between the two models.

**Results:**

Across patient cases, RBE‐weighted dose distributions from MCF MKM and mMKM showed strong agreement. Differences in target coverage were small, with CTV D95% differing by less than 1.6% across all disease sites and maximum target dose differences not exceeding 0.88%. Organ‐at‐risk dose deviations were limited, with differences of 3.0% or less across evaluated DVH metrics. Spatial dose‐difference maps showed that the largest discrepancies occurred in regions of steep dose gradients near target to organ‐at‐risk interfaces, while overall dose conformity and plan quality remained comparable between the two models.

**Conclusions:**

This study served as the first systematic model comparison of the MCF MKM and mMKM within a treatment planning environment. These findings suggest that the MCF MKM and mMKM produce dosimetrically consistent RBE‐weighted dose predictions under realistic planning conditions using carbon‐reference parameters. Accordingly, the fractionation schemes developed from years of clinical experience with mMKM implementations may serve as a practical foundation for protocol development at MCF.

## 1 INTRODUCTION

Modeling the relative biological effectiveness (RBE) is a fundamental aspect in treatment planning for carbon ion radiotherapy (CIRT), where the biological impact of radiation varies significantly with linear energy transfer (LET), tissue type, and dose per fraction.^1^, ^2^, ^3^, ^4^, ^5^, ^6^, ^7^, ^8^ Accurate RBE modeling is important for translating the physical dose of carbon ions into predictable biological outcomes. The two most widely implemented models for RBE calculation in CIRT are the Local Effect Model version I (LEM‐I)^9^, ^10^, ^11^ and the modified Microdosimetric Kinetic Model (mMKM).^12^ Both models have been integrated into commercial and open‐source treatment planning systems. While these models have been essential for clinical deployment of CIRT in Europe^13^, ^14^, ^15^ and Asia,^16^, ^17^, ^18^ their assumptions and biological foundations differ, resulting in notable variations in predicted RBE‐weighted dose distributions and clinical outcomes.^19^, ^20^, ^21^, ^22^, ^23^, ^24^ Reported differences between LEM‐I and mMKM can exceed 10% in target dose and significantly affect normal tissue complication probability estimates,^20^, ^25^ highlighting the clinical importance of model selection and validation.

The Mayo Clinic Florida Microdosimetric Kinetic Model (MCF MKM) was developed to provide an alternative RBE framework by integrating radiobiological characteristics of individual cell lines with microdosimetric input.^26^ Unlike the mMKM, which relies on microdosimetric quantities calculated using the Kiefer–Chatterjee radial dose distribution,^27^, ^28^, ^29^ MCF MKM employs an analytical microdosimetric function^30^, ^31^ created to quickly reproduce the results of track structure simulations in order to compute radiation quality parameters directly. In addition to this microdosimetric formulation, the MCF MKM was designed so that its biological parameters can be derived from photon rather than ion‐specific survival measurements, enabling the model to be applied predictively across different ion species and cell lines. The MCF MKM formulation also allows both linear and quadratic components of the cell‐survival response to vary with radiation quality, in contrast to the traditional mMKM implementation in which the quadratic term is treated as constant. Recent studies have shown that this quadratic behavior may depend on factors such as LET, depth, and dose rate, providing additional motivation for models that incorporate such variability.^32^ Previous validation studies demonstrated good agreement between MCF MKM predictions and experimental clonogenic survival data across multiple cell lines.^33^ Recent work established a pencil‐beam implementation of MCF MKM with Monte Carlo validation on clinical CT data.^34^

Despite growing evidence of its potential,^26^ the MCF MKM has not been directly compared against the mMKM using identical beam data and patient anatomy in a treatment planning environment. Such comparison is important because differences in the underlying microdosimetric formalism can quantitatively affect RBE and subsequent biological dose distributions. Previous work has shown that substituting one microdosimetric formulation for another within the same RBE framework, without appropriate parameter tuning, can substantially alter biological dose predictions.^27^, ^35^ Therefore, direct comparison under identical beam and patient‐geometry conditions is needed to quantitatively assess whether the analytical microdosimetric approach used in MCF MKM produces dosimetrically consistent treatment plans with the decades‐established mMKM framework under realistic planning conditions. Such comparison also helps to determine whether the extensive clinical experience and dose‐fractionation protocols developed at the National Institute of Radiological Sciences within the National Institutes for Quantum Science and Technology (NIRS‐QST)^36^, ^37^ in Japan using the mMKM can inform protocol development at MCF. The MCF MKM will serve as the clinical RBE model at the first CIRT center in the United States, currently under development at Mayo Clinic Florida (MCF).

Open‐source platforms such as *matRad*,^38^, ^39^ provide an ideal and flexible framework for such comparisons. *matRad* supports radiation therapy plan optimization with photons, protons and carbon ions, enabling DICOM import, biological dose optimization, and implementation of custom RBE models through integration of physical and biological base data.^38^, ^40^, ^41^

In this study, we present a systematic comparison of the MCF MKM and the mMKM using *matRad* with three specific objectives: (1) implement MCF MKM in *matRad* using Monte Carlo‐derived base data from the MCF carbon beamline, (2) compare RBE‐weighted dose distributions and dose metrics between MCF MKM and mMKM for multiple disease sites, and (3) assess whether NIRS‐QST clinical protocols can provide a foundation for treatment planning at MCF when using the MCF MKM model. We modeled the carbon ion beamline under development at MCF using the Tool For Particle Simulation (TOPAS),^42^, ^43^ a Monte Carlo platform based on Geant4,^44^, ^45^, ^46^ and extracted physical and biological base data for integration into *matRad*. Treatment plans were optimized on MCF patient data sets using the MCF MKM for representative clinical cases, following beam configuration and dose prescriptions from Yagi et al.^47^ RBE‐weighted doses were then recalculated using the mMKM with the same physical dose distribution, enabling direct model comparison. This work provides the first comprehensive evaluation of MCF MKM performance in a clinically realistic treatment planning framework and establishes a foundation for model‐informed CIRT protocol development at MCF.

## 2 MATERIALS AND METHODS

### 2.1 Overview

**FIGURE 1:**
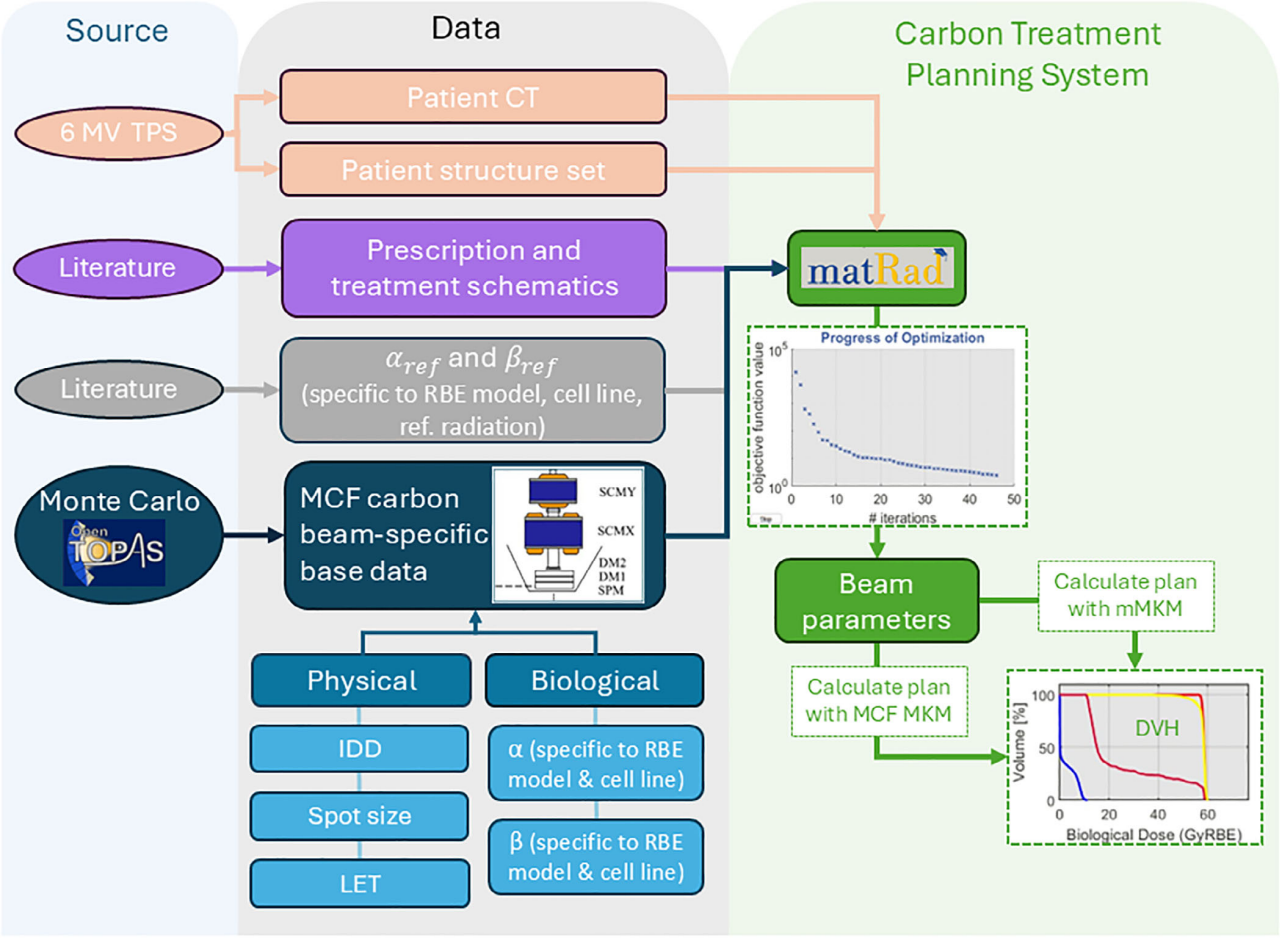
Schematic overview of the methodology applied to implement the MCF carbon ion machine within *matRad* and compare RBE models in a clinically realistic treatment planning framework. After machine implementation, *matRad* was used to optimize biological dose using the MCF MKM model, after which plans were recalculated using identical physical dose distributions with the mMKM model. Resulting dosimetric endpoints were compared.

This study compares the MCF MKM against the clinically established mMKM within a realistic treatment planning framework, the methodology of which is described in Figure 1. The MCF MKM was implemented in *matRad*, an open‐source treatment planning platform, using base data generated from Monte Carlo simulations of the MCF carbon beamline in TOPAS. Treatment plans were first optimized using the MCF MKM and subsequently recalculated using the mMKM, allowing for a direct comparison of the two RBE models on the same physical dose distribution. Optimizing with both models would result in different underlying physical dose distributions, making it impossible to attribute differences in RBE‐weighted dose solely to model behavior. By optimizing once with the MCF MKM and recalculating with both models, we isolated the effect of the RBE model itself while holding the physical dose constant. Although robust optimization is a standard element of clinical CIRT treatment planning,^48^, ^49^, ^50^, ^51^, ^52^ it was not incorporated in this initial comparative study as its purpose was to isolate differences between the two RBE models under identical physical dose conditions.

Physical and biological dose calculations for both models were performed using identical beam data to ensure consistency. Optimized plans were then evaluated for representative clinical cases to assess differences in RBE‐weighted dose distributions, target coverage, and organ‐at‐risk (OAR) sparing. This study extends previous MCF MKM water phantom evaluations^26^, ^33^, ^53^ to full patient data sets, allowing for model comparison under conditions representative of clinical treatment planning and further supporting the clinical adoption of the MCF MKM for CIRT at MCF.

### 2.2 Patient selection

Patients were selected from the MCF institutional database^54^ under an IRB‐approved protocol for retrospective research. Selection criteria were based on eligibility requirements defined for CIRT candidacy in the United States. Six disease sites were evaluated: prostate, pancreas, liver, lung, rectum, and bone sarcoma. The corresponding planning and delivery parameters for each site were modeled consistently with the Yagi et al.^47^ study and are summarized in Table 1. Normal tissue dose‐constraints were assigned using QUANTEC.^55^, ^56^ Treatment plans were generated in *matRad* using the MCF machine model, the description is provided in the following sections. All treatment plans were evaluated to ensure they met clinical quality criteria, defined as at least 95% of the target dose delivered to 95% of the clinical target volume (CTV), while respecting OAR constraints. Plans failing to meet these criteria were iteratively reoptimized with adjusted objective function weights until acceptable quality was achieved.

**TABLE 1:** Parameters used to optimize the patient treatment plan for each disease site.

					Fractions per beam			
Disease site	CTV volume [cc]	Prescribed dose	No. beams	No. beams per fraction	Beam 1	Beam 2	Beam 3	Beam 4
Liver	10.17	4 × 15 Gy(RBE) = 60.0 Gy(RBE)	3	2	2	2	4	–
Lung	271.8	4 × 15 Gy(RBE) = 60.0 Gy(RBE)	4	2	2	2	2	2
Pancreas	106.0	12 × 4.6 Gy(RBE) = 55.2 Gy(RBE)	4	1	3	3	3	3
Prostate	181.8	12 × 4.3 Gy(RBE) = 51.6 Gy(RBE)	2	1	6	6	–	–
Rectum	315.9	16 × 4.6 Gy(RBE) = 73.6 Gy(RBE)	3	1	7	3	6	–
Sarcoma	67.94	16 × 4.4 Gy(RBE) = 70.4 Gy(RBE)	3		5	5	6	–

### 2.3 MCF matRad machine

#### 2.3.1 Monte Carlo simulations

Base data were generated for the simulated MCF carbon beam using version 4.0 of the OpenTOPAS Monte Carlo simulation toolkit,^42^, ^43^ which is based on Geant4 version 11.1.3.^44^, ^45^ A cylindrical water phantom with a 5 cm radius was modeled, with monoenergetic carbon beams incident along the cylinder's central axis. Nominal beam energies ranged from 100 to 430 MeV/u, corresponding to water‐equivalent ranges of approximately 2–30 cm.

To optimize scoring resolution for subsequent integration with *matRad*, the phantom was divided into three longitudinal regions: the entrance region, Bragg peak region, and distal fragmentation tail. The entrance region length increased with beam energy (0.3–28 cm), while the Bragg peak and distal tail regions were fixed at 4 and 8 cm, respectively, to ensure consistent data capture extending 10 cm beyond the Bragg peak. Scoring resolution was energy‐dependent in the entrance region, ranging from 0.01 cm at low energies to 0.7 cm at high energies, maintaining a consistent number of bins across energies (depicted in Figure S1.1). Although this results in narrower entrance‐region bins than Bragg peak bins for some low‐energy beams, the entrance region exhibits relatively smooth depth‐dose variation compared with the Bragg peak, and the fixed high‐resolution Bragg peak scoring was prioritized to ensure consistent characterization of the peak and distal falloff while maintaining consistent data format. Resolution in the Bragg peak and distal tail regions was fixed at 0.05 and 1 cm, respectively.

A triangular energy distribution was used to model the source with no angular distribution. The spot size mirrored that specified for the MCF carbon nozzle. The following TOPAS modules were employed for all simulations: “g4em‐standard_opt4” “g4h‐phy_QGSP_BIC_HP” “g4decay” “g4ion‐binarycascade” “g4h‐elastic_HP” “g4stopping” following recommendations for particle therapy applications.^57^ Each simulation used 10^6^ primary particles. For all Monte Carlo simulations, a range cut of 0.05 mm was applied to all particle types when scoring physical quantities. A modified electron range cut was used exclusively for biological parameter scoring, as described in Section 2.3.3.

#### 2.3.2 Calculation of physical parameters

To calculate integrated depth‐dose (IDD) curves, the water phantom was binned along the beam axis (Z‐direction only), and the “DoseToMedium” scorer in TOPAS was used to determine dose deposition per slice. The unrestricted LET in water was calculated per slice using a custom TOPAS extension that interpolated mass stopping powers as functions of ion energy and atomic number, using ICRU Report 90 for ^1^H and ^4^He ions (I = 78 eV) and Errata and Addenda: ICRU Report 73 for all other scored ions (I = 75 eV).^58^, ^59^ The LET contributions by each particle were weighted by dose. These scored outputs were input into the *matRad* MCF machine as a function of the midpoint of the scoring bin.

To quantify spot size as a function of depth, a parallel world geometry was implemented to introduce additional radial binning at 0.2 mm intervals, while preserving the variable longitudinal binning along the beam axis. At each Z‐position, the radial dose distribution was fit to a single Gaussian using MATLAB R2023a to extract the lateral width (σ). The resulting IDD, LET, and lateral distribution data were assigned to the midpoint of each scoring slice and imported into the matRad MFC machine model.

#### 2.3.3 Calculation of biological parameters

Biological parameters required to compute RBE were calculated using the mMKM and MCF MKM. These models are each based on the linear‐quadratic model of cell survival,^60^, ^61^ and can be calculated as a function of absorbed dose to water (D) using

(1)

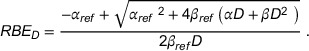




Here, $\alpha_{ref}$ and $\beta_{ref}$ are the terms of the linear quadratic model for the reference radiation and α and β the corresponding parameters for the ion beam. The latter quantities were directly scored in TOPAS using the following methods. As detailed elsewhere, the clinical dose was then calculated as the product between the absorbed dose, the *RBE_D_* and a clinical scaling factor.

In order to implement mMKM in *matRad*, a table of saturation‐corrected dose mean specific energy (${z_{D}}^{*}$) as a function of ion kinetic energy was created in‐house and validated against previous work.^27^ The quantity ${z_{D}}^{*}$ is a microdosimetric metric that incorporates biological parameters in order to account for the overkill effect of cell survival at high LET, where the increased density of energy deposition results in a decreased efficiency of cell killing per unit of dose.^62^, ^63^, ^64^ This table was derived using the Kiefer–Chatterjee radial dose distribution, implemented for ions incident on a cylindrical target whose length equals its diameter.^27^, ^65^, ^66^ A custom TOPAS extension was written to score the mixed‐field ${z_{D}}^{*}$ as a function of depth for each monoenergetic carbon beam, weighting the contribution from each ion (i) depositing the dose $D_{i}$ in the corresponding voxel using

(2) $${z_{D}}^{*}=\frac{\sum_{i}{z_{D,i}}^{*}\cdot D_{i}}{\sum_{i}D_{i}}\;.$$

Following the mMKM formalism^12^ α was calculated as:

(3) $$\alpha =\alpha_{0}+\beta {z_{D}}^{*},$$

where $\alpha_{0}$ corresponds to the value of α in the limit of LET approaching zero. The TOPAS scoring of ${z_{D}}^{*}$ required specification of the radii of both the subnuclear domain in which lethal events are assumed to occur ($r_{d})$ and the cell nucleus ($R_{n})$. For the application of mMKM in this study, the following reference parameters were used^28^: $\alpha_{0}=0.172\;Gy^{-1},\;\;r_{d}=0.32\;\mu m,\;\;R_{n}=3.9\;\mu m.$ Our mMKM *matRad* base data was validated against the published model implementation by Yoon et al.^40^

For the MCF MKM,^26^, ^33^ α and β were scored using a validated TOPAS extension^53^ that integrates an analytical microdosimetric function^30^ to calculate the microdosimetric spectra for each ion incident on the voxel. From the microdosimetric spectra, α and β are calculated from the lineal energy (y) according to the following equations^26^, ^33^:

(4) $$\alpha =\int \left(\alpha_{0}+\beta_{0}\frac{y}{\rho \pi {r_{d}}^{2}}\right)\;c\left(y\right)d\left(y\right)dy$$

and

(5) $$\beta =\beta_{0}{\left[\int c\left(y\right)d\left(y\right)dy\right]}^{2}\;,$$

where

(6) $$c\;\left(y\right)=\frac{1-exp\left[-\left(\alpha_{0}+\beta_{0}\frac{y}{\rho \pi {r_{d}}^{2}}\right)\left(\frac{y}{\rho \pi {R_{n}}^{2}}\right)-\beta_{0}{\left(\frac{y}{\rho \pi {R_{n}}^{2}}\right)}^{2}\right]}{\left(\alpha_{0}+\beta_{0}\frac{y}{\rho \pi {r_{d}}^{2}}\right)\left(\frac{y}{\rho \pi {R_{n}}^{2}}\right)+\beta_{0}{\left(\frac{y}{\rho \pi {R_{n}}^{2}}\right)}^{2}}.$$

Therefore, d(y) represents the relative dose contribution from lineal energy events, whereas c(y) modifies this contribution to account for saturation effects at high local energy deposition. These parameters were then weighted according to the dose contribution from each ion incident on a voxel to calculate mixed field α and β using the following^67^

(7) $$\alpha =\frac{\sum_{i}D_{i}\cdot \alpha_{i}}{\sum_{i}D_{i}}\;,$$

and

(8) $$\beta ={\left(\frac{\sum_{i}D_{i}\cdot \sqrt{\beta_{i}}}{\sum_{i}D_{i}}\right)}^{2}\;.$$

The MCF MKM parameters for the HSG cell line were previously derived from the photon dose‐response and measurable cell characteristics: $R_{n}=4.5\;\mu m$, $r_{d}=0.28\;\mu m$, $\alpha_{0}=0.117\;Gy^{-1},$ and $\beta_{0}=0.0615\;Gy^{-2}$.^26^ Details regarding the microdosimetric function and the model calculation can be found in previous works.^26^, ^53^

As required for the use of the analytical microdosimetric function, an artificially high electron range cut (10 m) was applied exclusively during Monte Carlo calculations of α and β to force all electrons to deposit their energy locally and avoid double‐counting their contributions to the microdosimetric distributions. The validity and quantitative impact of this approach have been previously evaluated in detail.^30^

In the clinical implementation of the LEM‐I, the reference radiation is always photon‐based, reflecting its derivation from x‐ray irradiation data. In contrast, the mMKM historically used at the NIRS‐QST employs carbon as the reference radiation, with clinical dose prescriptions scaled according to a factor of 2.41.^28^, ^35^ The MCF implementation of the MKM (i.e., the MCF MKM) will similarly adopt a carbon ion reference when the carbon beamline becomes clinically operational. Accordingly, all RBE models in this work were implemented in *matRad* using carbon‐reference values derived from the midpoint of a 6 cm wide SOBP at the NIRS‐QST (range = 21 cm). The radiation quality at the center of this SOBP was used as the reference radiation for RBE computation, consistent with the clinical convention established for the mMKM framework. The corresponding reference parameters were: for MCF MKM $\alpha_{ref,C}=1.019\;Gy^{-1}$ and $\beta_{ref,C}=0.0498Gy^{-2},$ and for mMKM $\alpha_{ref,C}=0.7554\;Gy^{-1}$ and $\beta_{ref,C}=0.0615Gy^{-2}$.^28^ As the dose prescriptions reported by Yagi et al. were defined relative to carbon as a reference,^47^ this approach enabled direct use of those prescriptions while preserving clinical relevance and consistency of each RBE model. For this study, per‐fraction prescription doses were first divided by 2.41 to obtain the biological prescription. For cases delivered using multiple ports, including the lung and liver cases, this conversion was applied to the prescribed dose for each port. Plans were optimized to this prescription, and the resulting RBE‐weighted dose distributions were then multiplied by 2.41 to yield the clinical dose, consistent with the mMKM carbon‐reference convention:

(9) $$D_{clin}=RBE\cdot D\cdot 2.41$$

For evaluation over the total plan, clinical dose per‐fraction and per‐port, where applicable, were summed across all factions.

### 2.4 matRad optimization

Prior to clinical plan optimization, water phantom plans were optimized and calculated on cubic targets to validate both the implementation of the RBE models and the configuration of the MCF carbon machine in *matRad*. Configurations were carefully selected for comparison with previously published and in‐house Monte Carlo calculations.^28^, ^35^ This validation ensured consistency between the RBE model implementation and physical beam data before applying the framework to patient‐specific treatment plans.

Each treatment field was optimized independently with the MCF MKM following the beam configurations described by Yagi et al.,^47^ with gantry angles in *matRad* set to reproduce the corresponding beam angle, patient orientation, and table position reported in that study. Clinical dose was calculated for each field using each of the MCF MKM and mMKM, and subsequently summed across beams. For target coverage, a squared underdosing objective was applied to the planning target volume (PTV), penalizing voxels receiving doses below the prescription dose quadratically in proportion to the deviation from the prescribed value. To limit excessive dose, a squared overdosing objective was additionally applied above 110% of the prescribed dose, penalizing hotspots beyond this threshold. No plan normalization was performed; instead, absolute dose objectives were used directly in the optimization. The optimization typically converged within 300–500 iterations, depending on the number of beams and model complexity.

All dose calculations were performed on a 3 mm^3^ dose grid using *matRad* “Cleve” v3.1.0 with MATLAB 9.14. For the lung and liver treatment sites, the prescribed doses per fraction exceeded 5 Gy(RBE), which surpasses the hard‐coded upper limit in *matRad* associated with the LEM implementation. This restriction was overridden for the purpose of this study.

## 3 RESULTS

Physical and biological base data calculated using TOPAS for the MCF machine in *matRad* for each of the 161 carbon ion beams are shown in Figures S1.2 and S1.3. These datasets include depth‐dose and LET distributions, along with model specific α and β distributions that serve as the foundation for clinical dose optimization in *matRad*.

To validate the implementation of the RBE models, clinical dose was first optimized and recalculated in homogeneous cubic water phantoms, an example of which can be seen in Figures S2.1 and S2.2 for parallel opposed beams optimized to a dose of 5 Gy(RBE) per fraction. The phantom configurations spanned the range of dose prescriptions that were used in this study, and were designed to match previously published SOBP simulations and in‐house Monte Carlo calculations^28^, ^35^ to evaluate the models under controlled physical conditions before application to patient geometries. For each model, the line profiles of calculated α, β, RBE, and biological dose were consistent with the corresponding reference Monte Carlo results in all planes. In addition, the biological dose distributions calculated using the MCF MKM and mMKM models agreed within 1.5% across the water phantom. This validation confirmed that both model implementations produced expected RBE‐weighted dose behavior at dose levels consistent with the clinical prescriptions adopted in this study.

**FIGURE 2:**
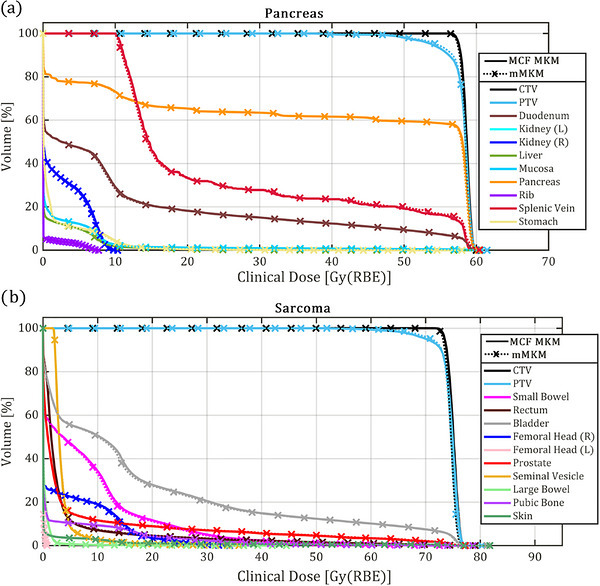
Clinical DVH comparisons of MCF MKM and mMKM RBE models for the: (a) pancreas and (b) sarcoma disease sites.

**FIGURE 3:**
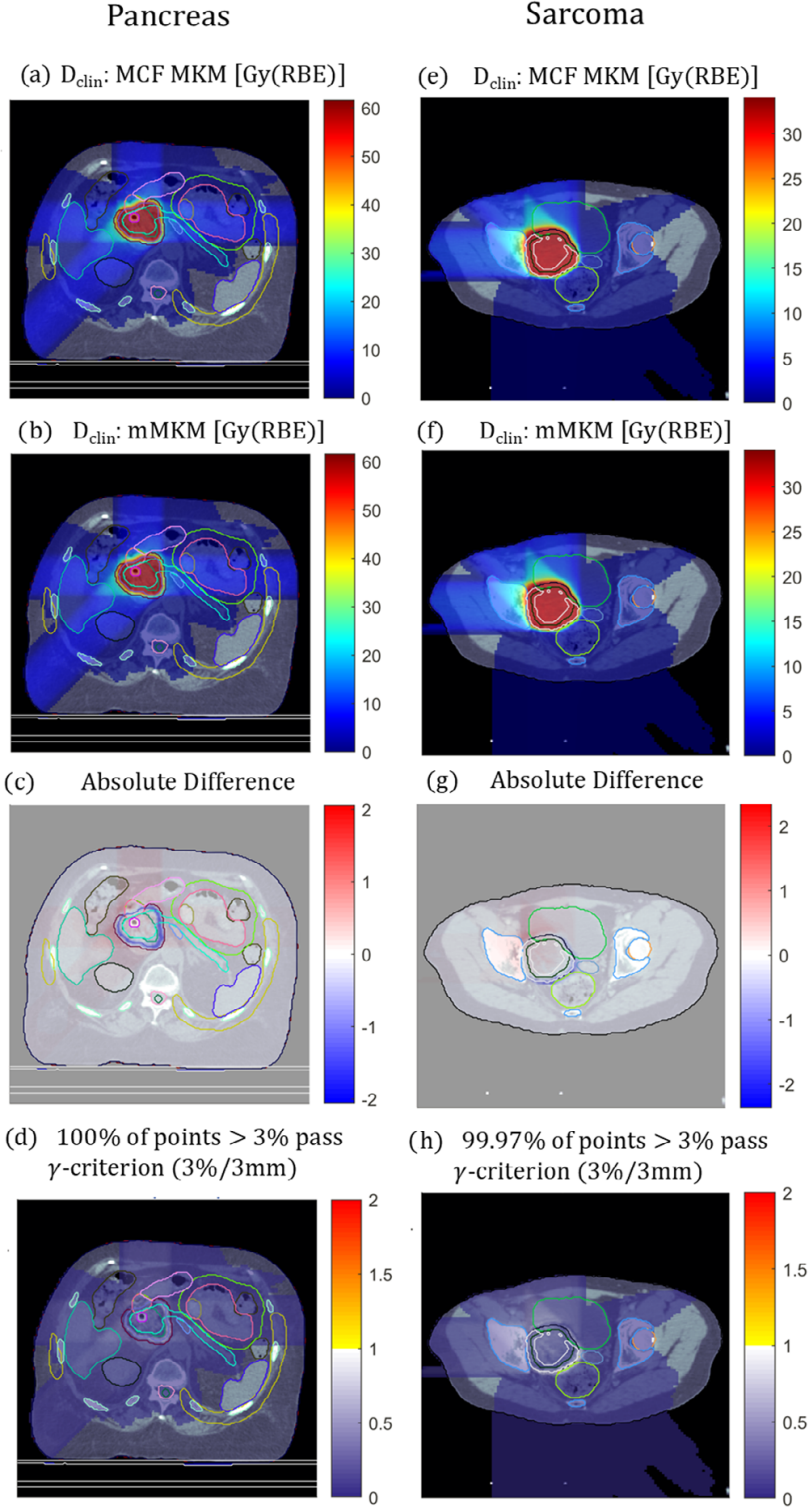
Clinical dose distributions optimized using MCF MKM and calculated with each MCF MKM (a and d) and mMKM (b and e) for: (a–d) pancreas and (e–h) sarcoma disease sites. The absolute difference in clinical dose is shown (c and g), along with gamma pass rates (d and h).

DVHs for two of the six disease sites investigated in this study, pancreas and pelvic bone sarcoma, are presented in Figure 2. The DVHs display clinical dose distributions calculated using both the mMKM and MCF MKM models, illustrating their close agreement across the target and OARs. Corresponding treatment plans are shown in Figure 3, which illustrate the clinical dose distributions calculated for each RBE model and the absolute voxel‐wise differences between them in the form of heat maps. These visualizations highlight the spatial distribution of clinical dose and demonstrate that the largest differences between the two models occur primarily in OARs immediately adjacent to the target, where steep dose gradients are present. A heat map of the gamma‐criterion (3%/3 mm) pass rates are also shown in Figure 3 for the pancreas and sarcoma sites. With these criteria, gamma analysis across all sites had a pass rate of 99%, in both PTV and OAR structures. DVHs for the remaining four sites (lung, liver, prostate, and rectum) are provided in Figure S2.3.

Table 2 summarizes the quantitative evaluation metrics for all treatment sites, listing the targets and primary OARs for each plan together with their respective constraint parameters. For each structure, the table reports the corresponding dose or volume values derived from the clinical dose distributions of the MCF MKM and mMKM, as well as the percent difference between the two. Across all cases, the clinical dose difference between RBE models reached up to 0.88% for targets and 3.0% for OARs, with mean differences of 0.41% and 1.2%, respectively. Target evaluations were performed using the CTV, with D95% extracted as the representative metric. The D95% coverage ranged from 3% to 4% higher than that of the prescribed dose across all treatment sites, indicating consistent and clinically acceptable target coverage. Overall, the results confirm strong agreement between MCF MKM and mMKM clinical dose predictions, with only minor deviations observed in high‐gradient regions near interfaces between targets and OARs.

**TABLE 2:** DVH parameter summary by treatment site, comparing clinical doses from MCF MKM and mMKM models.

Disease site	Structure	Dose constraint	MCF MKM	mMKM	Percent difference
Liver	CTV	D95%	62.4 Gy(RBE)	62.1 Gy(RBE)	−0.43%
	Liver	V40	0.37%	0.38%	2.67%
	Esophagus	D2%	0.01 Gy(RBE)	0.01 Gy(RBE)	0.00%
	Bowel	D2%	14.6 Gy(RBE)	14.8 Gy(RBE)	1.09%
	Spinal Canal	D2%	0.16 Gy(RBE)	0.16 Gy(RBE)	0.00%
Lung	CTV	D95%	62.8 Gy(RBE)	62.6 Gy(RBE)	−0.37%
	Lung (total)	V40	7.58%	7.52%	−1.06%
	Trachea	D2%	1.69 Gy(RBE)	1.64 Gy(RBE)	−3.00%
	Spinal cord	D2%	2.82 Gy(RBE)	2.87 Gy(RBE)	1.76%
Pancreas	CTV	D95%	57.6 Gy(RBE)	57.4 Gy(RBE)	−0.28%
	Stomach	D2%	12.1 Gy(RBE)	11.8 Gy(RBE)	−2.68%
	Bowel	D2%	8.61 Gy(RBE)	8.45 Gy(RBE)	−1.88%
	Spinal cord	D2%	0.16 Gy(RBE)	0.16 Gy(RBE)	0.00%
	Kidney left	D2%	0.00 Gy(RBE)	0.00 Gy(RBE)	0.00%
	Kidney right	D2%	8.90 Gy(RBE)	8.71 Gy(RBE)	−2.16%
Prostate	CTV	D95%	53.3 Gy(RBE)	53.2 Gy(RBE)	−0.09%
	Bladder	V40	3.47%	3.49%	0.57%
	Bowel	D2%	0.00 Gy(RBE)	0.00 Gy(RBE)	0.00%
	Femoral head (L)	D2%	20.2 Gy(RBE)	20.0 Gy(RBE)	−1.00%
	Femoral head (R)	D2%	20.2 Gy(RBE)	20.0 Gy(RBE)	−0.95%
Rectum	CTV	D95%	76.7 Gy(RBE)	76.1 Gy(RBE)	−0.88%
	Bowel	D2%	20.6 Gy(RBE)	20.5 Gy(RBE)	−0.39%
	Femoral head (L)	D2%	3.85 Gy(RBE)	3.91 Gy(RBE)	1.55%
Sarcoma	CTV	D95%	73.5 Gy(RBE)	73.2 Gy(RBE)	−0.42%
	Bowel	D2%	23.4 Gy(RBE)	23.1 Gy(RBE)	−1.34%
	Cauda equina	D2%	42.6 Gy(RBE)	41.7 Gy(RBE)	−2.09%
	Skin	D2%	0.00 Gy(RBE)	0.00 Gy(RBE)	0.00%

## 4 DISCUSSION

This study demonstrated close agreement between the MCF MKM and mMKM across all evaluated carbon ion treatment plans, with RBE‐weighted dose differences up to 1.4% for target volumes and 3.0% for OARs. Target coverage metrics (D95%) differed by less than 1.6% across all six disease sites, while dose constraints for critical normal tissues were met with comparable margins using both models (Table 2). These findings indicate that despite their differing mathematical and microdosimetric formalism, the analytical microdosimetric function in MCF MKM versus Kiefer–Chatterjee radial dose distribution in mMKM, the two models produce dosimetrically consistent predictions when applied to realistic patient anatomy and beam delivery configurations.

The observed agreement between MCF MKM and mMKM has important implications for clinical implementation at MCF. The close correspondence in RBE‐weighted dose distributions suggests that the dose fractionation schemes and clinical protocols established at NIRS‐QST over decades of clinical experience may provide a reasonable starting point for protocol development at the first American CIRT center. Furthermore, while the models agree under the planning conditions tested here, their responses to different fractionation may be different and would warrant a prospective clinical validation. The deviations between the results of two models are expected to be larger at higher doses (i.e., for hypofractionated or single fraction lung patient) due to the models’ different handling of the quadratic term of cell survival (*β*), which is radiation quality independent in the mMKM, while radiation dependent in the MCF MKM.^26^ This limitation of the mMKM had been solved by introducing the stochastic microdosimetric kinetic model as described in Inaniwa and Kanematsu.^68^

Although this study did not include recalculation of the RBE‐weighted dose distribution using Monte Carlo (TOPAS), future work will incorporate such validation to comprehensively evaluate the MCF MKM implementation against full simulations. However, significant deviations are not expected based on prior benchmarking of the mMKM in *matRad*, which demonstrated excellent agreement with TOPAS results.^40^ In that work, the RBE‐weighted dose distribution agreed within 4.0% between *matRad* and Monte Carlo calculations for an inhomogeneous phantom. Similarly, in patient cases, DVH parameters for the target volume differed by less than 2.7%, with range differences below 0.7%. Given these small discrepancies, comparable agreement is expected for the MCF MKM implementation presented here, as the underlying biophysical formulation and numerical framework are similar. Therefore, recalculation with TOPAS is not anticipated to dramatically alter the dosimetric or biological conclusions of this study, though it will be addressed in future work for completeness.

It is worth mentioning that both models used carbon ion reference radiation from the same NIRS‐QST SOBP^28^ and biological parameters derived from HSG cell line. These parameters are unchangingly applied for all tissues, tumors, and patients, and thus which may not fully represent the biological variability across them. Additionally, we evaluated a limited number of patient cases, which may not capture the full range of anatomical complexity and inter‐patient variability encountered in clinical practice. Despite this, the agreement observed across anatomically diverse sites suggests that increasing the number of patients is unlikely to impact the fundamental model‐to‐model trends. This work serves as proof of concept, providing the first step toward systematic evaluation of the MCF MKM within a clinical treatment planning framework. Future prospective studies will enable direct comparison of clinically deliverable treatment plans optimized with each RBE model, providing an evaluation of potential differences in dose distributions,

Our analysis used carbon ion reference radiation for both models, which differs from the photon‐based reference traditionally used with LEM‐I. This choice limits direct comparison with European clinical experience using LEM‐I. As numerous studies have already provided extensive comparisons of LEM‐I and mMKM across beam qualities, tissue types, and treatment sites,^24^, ^25^, ^69^, ^70^, ^71^, ^72^, ^73^ the present work focuses on validating the MCF MKM as a new implementation within *matRad*. Future studies will extend this comparison to include the LEM‐I model under consistent reference conditions, enabling a broader evaluation of model‐specific differences in RBE predictions. Together, these efforts will establish a more complete understanding of the predictive reliability and clinical applicability of the MCF MKM for CIRT planning.

## 5 CONCLUSIONS

MCF MKM demonstrated close agreement with mMKM across multiple disease sites when implemented in *matRad* using Monte Carlo‐derived base data from the MCF carbon beamline. RBE‐weighted dose distributions, target coverage metrics, and OAR dose constraints showed differences below 3.0%, suggesting that the two microdosimetric models produce dosimetrically equivalent clinical predictions under the planning conditions evaluated in this study. These findings indicate that the extensive clinical experience and protocols established at NIRS‐QST using mMKM can provide a reasonable foundation for protocol development at MCF, while recognizing that site‐specific commissioning, comprehensive uncertainty analysis, and prospective clinical validation remain essential for clinical implementation. This work provides initial evidence for the applicability of the MCF MKM framework within a treatment‐planning context and establishes a methodology for ongoing RBE model evaluation in CIRT planning.

## AUTHOR CONTRIBUTIONS

*Conceptualization, methodology, and formal analysis*: Keith M. Furutani, Sridhar Yaddanapudi, and Shannon Hartzell. *Software*: Shannon Hartzell, Niklas Wahl, Remo Cristoforetti, Sridhar Yaddanapudi, Lisa Seckler, and Alessio Parisi. *Validation*: Shannon Hartzell, Keith M. Furutani, Alessio Parisi, and Sridhar Yaddanapudi. *Investigation*: Shannon Hartzell, Sridhar Yaddanapudi, Alessio Parisi, and Keith M. Furutani. *Data curation*: Shannon Hartzell, Sridhar Yaddanapudi, and Alessio Parisi. *Project administration, supervision and funding acquisition*: Keith M. Furutani and Chris J. Beltran. *Writing and editing*: All authors. All authors have read and agreed to the submitted version of the manuscript.

## ETHICS STATEMENT

The study was approved by the Mayo Clinic Institutional Review Board (#23‐007695). This study complied with all applicable laws regarding participant privacy. The data used contains only de‐identified patient information (i.e., does not include name, addresses, social security or medical record numbers, or other obvious identifiers), and is fully compliant with HIPAA. Study results omitted subject identification; therefore, informed consent was not required. The publication does not include subject identifiers.

## CONFLICT OF INTEREST STATEMENT

The authors have no relevant conflicts of interest to disclose.

## Supporting information



Supporting Information

## Data Availability

The raw data required to reproduce the findings herein are available from the authors upon request.
