# Supplementary material for: Comparative evaluation of the Mayo Clinic Florida microdosimetric kinetic model and mMKM for carbon ion treatment planning: A matRad‐based analysis
Source: J Appl Clin Med Phys. 2026 May 31;27(5):e70645. doi: 10.1002/acm2.70645 (PMC13239107; doi:10.1002/acm2.70645)
Supplement: Supplementary file 1 — Supporting Information [file ACM2-27-e70645-s001.docx]

**Supplementary Material**

**Comparative Evaluation of the Mayo Clinic Florida Microdosimetric Kinetic Model and mMKM for Carbon Ion Treatment Planning: a matRad-based Analysis**

**SM1. *matRad* Base Data**

*SM1.1 Physical Base data*

To generate the base data required for RBE model development and integration into *matRad*, Monte Carlo simulations were performed in a homogeneous water phantom. The simulations were designed to produce depth-resolved physical and biological quantities realistic to the planned MCF carbon beamline, providing a consistent dataset for use in the *matRad* treatment planning system.


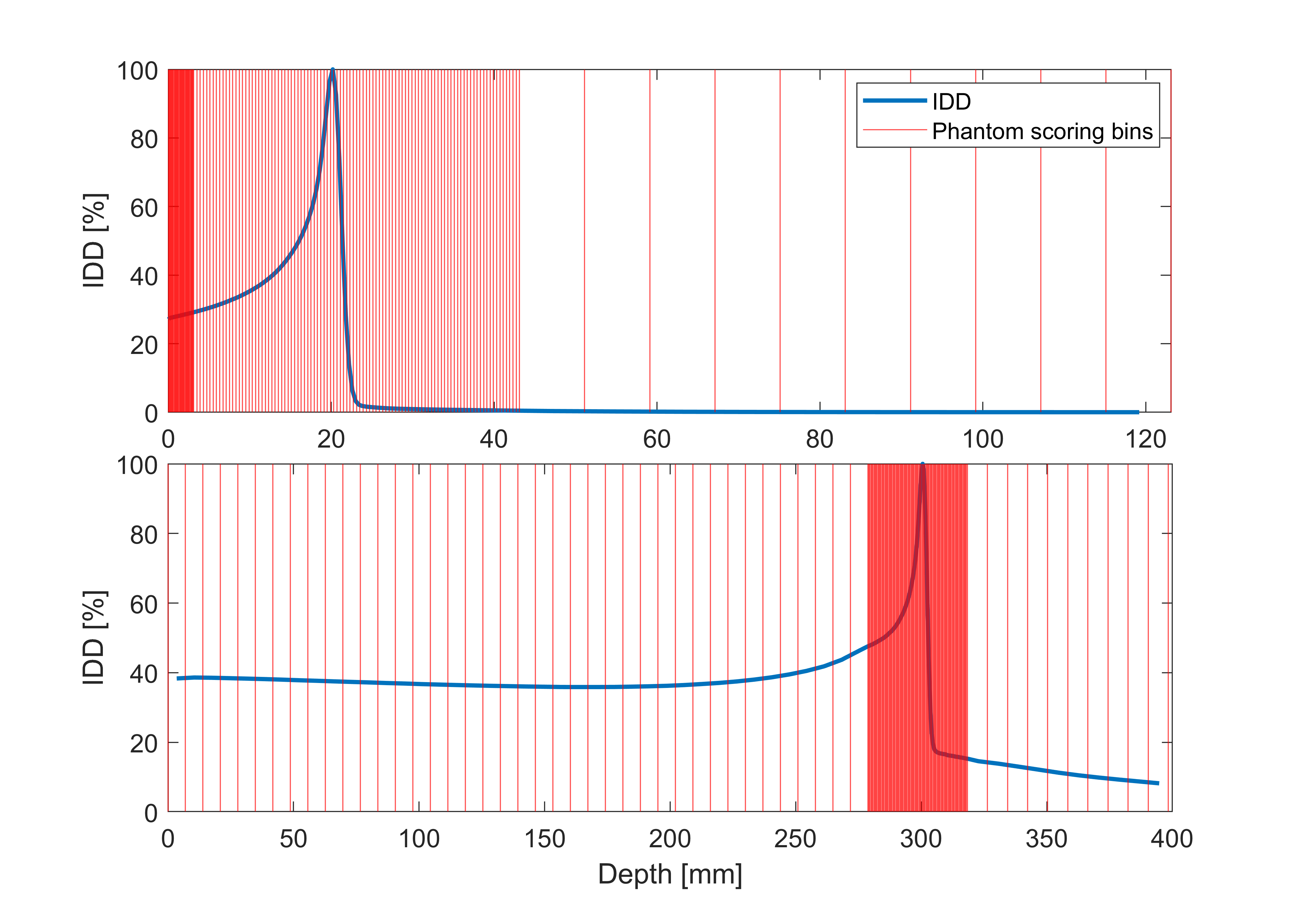
To optimize scoring resolution for subsequent integration with *matRad*, the phantom was divided into three longitudinal regions: the entrance region, Bragg peak region, and distal fragmentation tail. The entrance region length increased with beam energy (0.3–28 cm), while the Bragg peak and distal tail regions were fixed at 4 cm and 8 cm, respectively, to ensure consistent data capture extending 10 cm beyond the Bragg peak. Scoring resolution was energy-dependent in the entrance region, ranging from 0.01 cm at low energies to 0.7 cm at high energies, maintaining a consistent number of bins across energies. Resolution in the Bragg peak and distal tail regions was fixed at 0.05 cm and 1 cm, respectively. A diagram of the resolution can be seen in Figure SM-1.1 for 100 and 430 MeV/u carbon beams.

**Figure SM-1.1** IDD scored as a function of depth (blue) for each 100 MeV/u (top) and 430 MeV/u (bottom) carbon ion beams incident on a cylindrical water phantom (r=5 cm, variable length). The red bins indicate the scoring resolution in each of the three regions: entrance, Bragg peak, and fragmentation tail, as differentiated by the change in bin size.

The physical base data, including IDD and LET, scored in TOPAS and used as an input into *matRad* for this study is shown for each carbon ion energy as a function of depth in Figure SM-1.2.


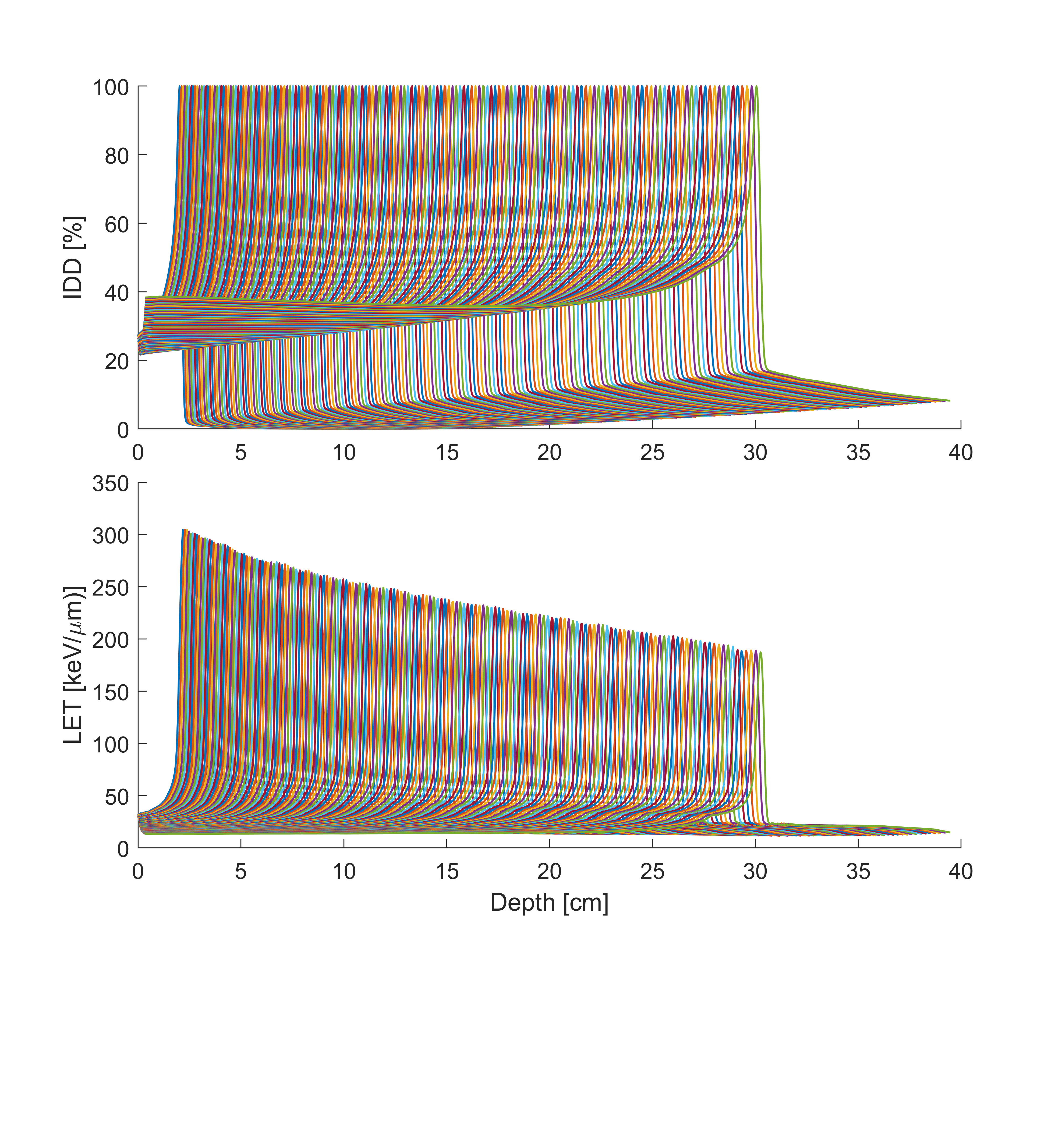


**Figure SM-1.2** IDD (top) and LET (bottom) calculated as a function of depth for each of the monoenergetic carbon beams scored in this study, ranging from 100 to 430 MeV/u at 2 MeV/u increments.

*SM1.2 Biological Base data*

The biological base data generated for this study are shown in Figure SM-1.3. In the MCF MKM, both the linear and quadratic components of the cell-survival response naturally vary with depth and radiation quality, resulting in β values that change across the Bragg curve. This behavior reflects recent studies indicating that the quadratic term may depend on LET, dose rate, and track-structure characteristics [29], and stands in contrast to the traditional mMKM implementation in which β is treated as constant. The patterns observed in Figure SM-1.3 therefore illustrate one of the key conceptual differences between the two models.

**
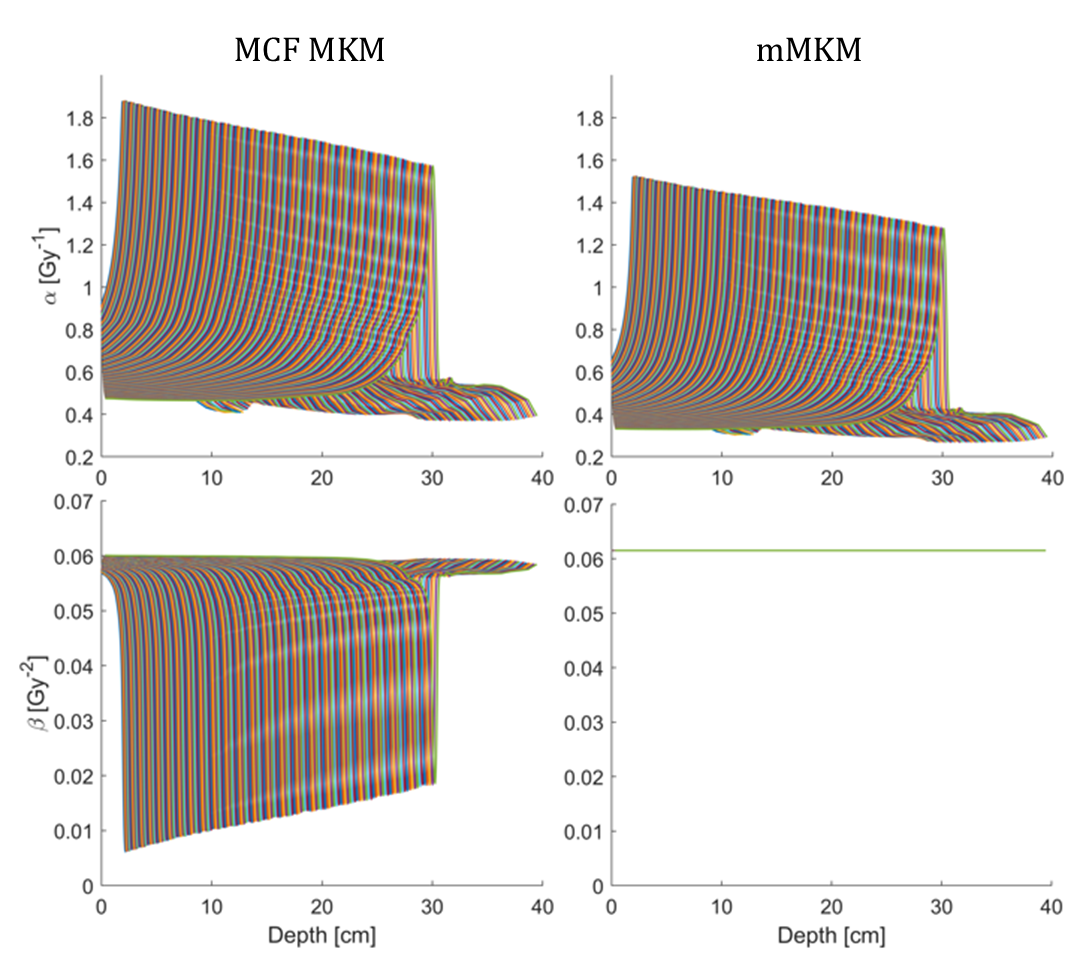
**

**Figure SM-1.3** Alpha (top) and beta (bottom) calculated as a function of depth for each of the monoenergetic carbon beams scored in this study, ranging from 100 to 430 MeV/u at 2 MeV/u increments. Values are presented for MCF MKM (left) and mMKM (right).

**SM2. Treatment Plans**

*SM2.1 Validation of Model Implementation*


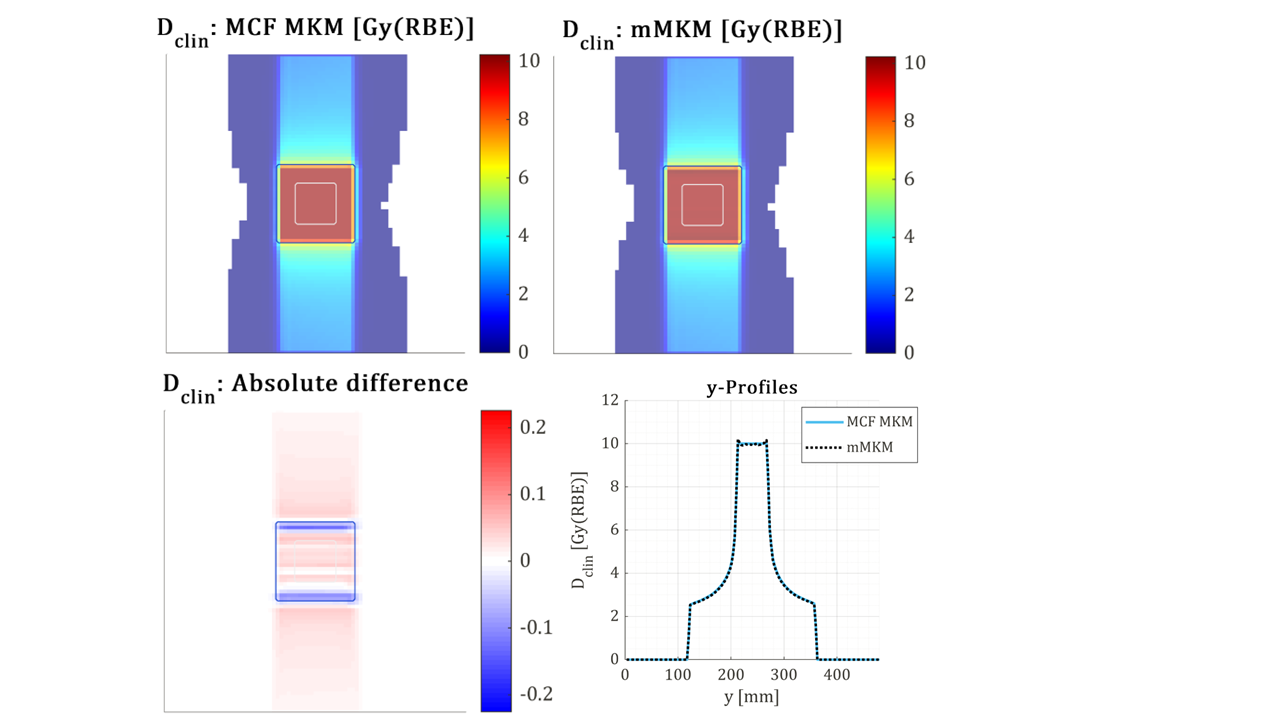
To validate the implementation of the RBE models in *matRad,* a CT scan of a water phantom was imported, and a treatment plan was generated for a 25 × 25 × 25 cm^3^ water cube (PTV). A clinical dose of 5 GyRBE was prescribed, and the plan was optimized to the PTV. An arbitrary CTV was defined within the PTV, where the resulting dose distributions calculated using MCF MKM and mMKM was compared with previous in-house and published Monte Carlo simulations for validation [25,63]. The treatment plan and associated line profiles of the clinical dose are shown in Figure SM-2.1, and corresponding DVH shown in Figure SM-2.2. The clinical dose distributions agreed within 1.5 % throughout the phantom, affirming the model implementation.

**Figure SM-2.1** Clinical dose distributions optimized using MCF MKM and calculated with each MCF MKM (a) and mMKM (b) for a water phantom. The absolute difference in clinical dose is also shown (c), along with the profile of biological dose along the beam axis (d).

**
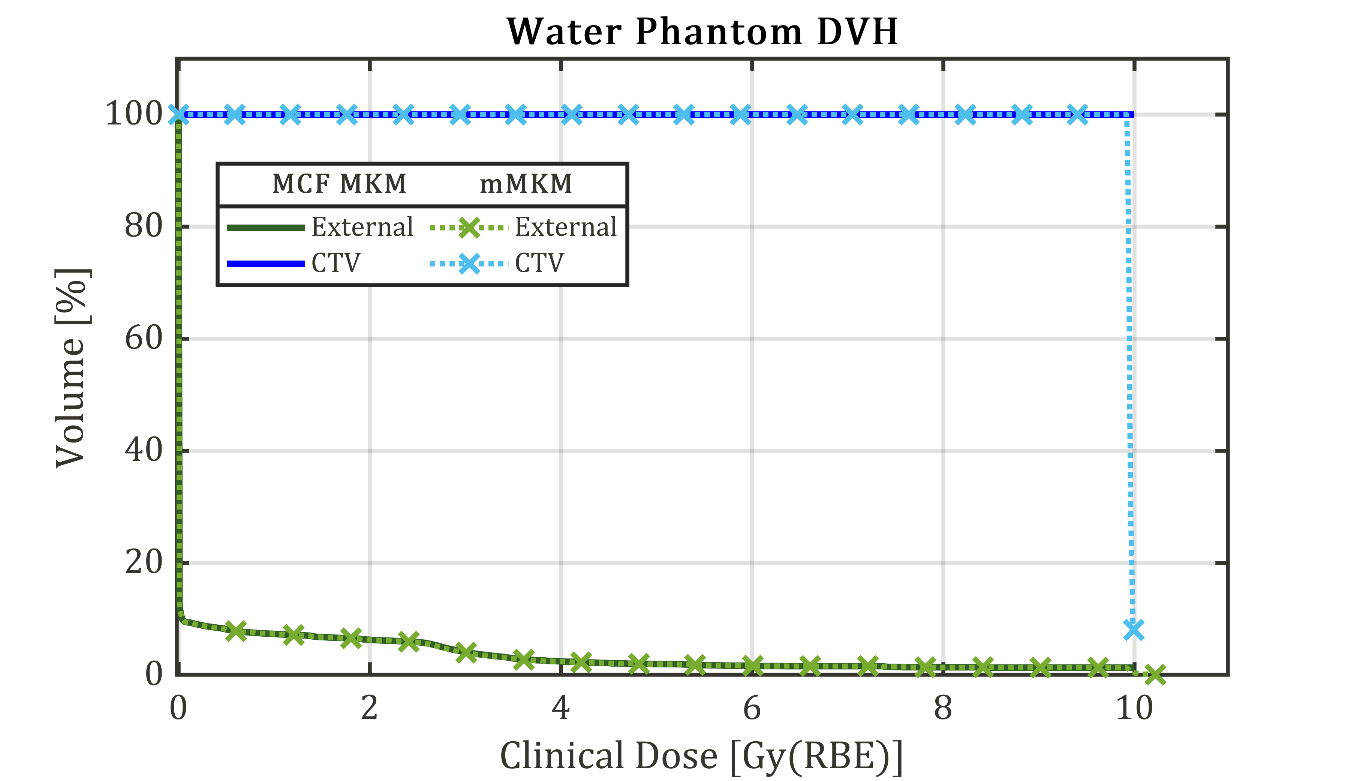
**

**Figure SM-2.2 C**linical DVH comparisons of MCF MKM and mMKM RBE models for a water phantom used for validation of RBE model implementation within matRad.

*SM2.2 Site-specific Treatment Plans*

Figure SM-2.3 presents the DVHs for the four treatment sites not included in the main text: lung, liver, prostate, and rectum. For each site, the biological dose distributions calculated using the mMKM and MCF MKM models are shown for both the targets and principal OARs. These results complement Figure 2 in the main text and further demonstrate the close agreement between the two RBE models across all clinical configurations examined.


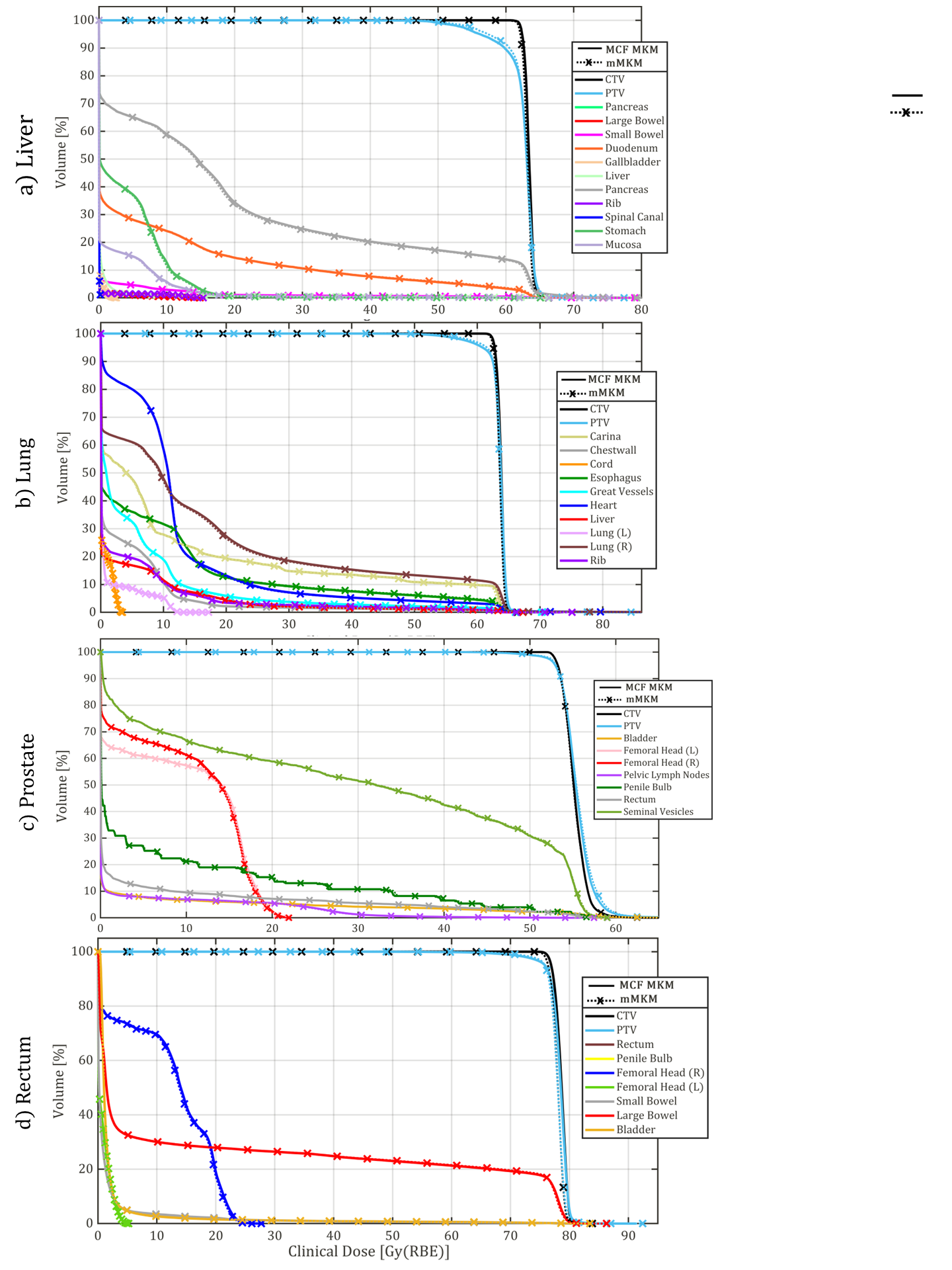


**Figure SM-2.3.** DVHs for the liver (a), lung (b), prostate (c), and rectum (d) treatment sites. Biological dose distributions were calculated using the mMKM and MCF MKM models for each target and associated organs at risk, demonstrating close agreement between the two RBE models across all cases.
